# Efficacy of medication reconciliation in medication therapy for chronic diseases in elderly patients with coronary atherosclerotic heart disease

**DOI:** 10.12669/pjms.41.9.11187

**Published:** 2025-09

**Authors:** Maodong Zheng, Naidong Wang, Xujun Hu, Juan Yan, Yuhuan Cui

**Affiliations:** 1 Maodong Zheng Department of Pharmacy, The First Affiliated Hospital of Hebei North University, Zhangjiakou 075000, Hebei, China; 2 Naidong Wang Department of Pharmacy, Ji Nan Hospital, Jinan 250013, Shandong, China; 3 Xujun Hu Dept. of Pharmacy, Peking University Third Hospital Chongli, Zhangjiakou 075000, Hebei, China; 4 Juan Yan Department of Pharmacy, The First Affiliated Hospital of Hebei North University, Zhangjiakou 075000, Hebei, China; 5 Yuhuan Cui Department of Gerontology, The First Affiliated Hospital of Hebei North University, Zhangjiakou 075000, Hebei, China

**Keywords:** Coronary atherosclerotic heart disease, Medication reconciliation

## Abstract

**Objective:**

To explore the service model and effect of pharmacist-conducted medication reconciliation (MR) in geriatric patients with coronary atherosclerotic heart disease (coronary heart disease, CHD), so as to provide a reference for grass-roots pharmacists in MR implementation.

**Methodology:**

This was a retrospective study. A total of 200 CHD patients admitted to the department of Geriatrics in the First Affiliated Hospital of Hebei North University from January 2023 to July 2024 were randomly divided into a control group (n = 100) and an experimental group (n = 100). The control group was given conventional clinical treatment, while the experimental group was additionally serviced with standardized pharmacist-conducted MR. Both groups were followed up till six months after discharge. PIM use during hospitalization, length of hospital stay, drug costs, incidence of ADRs, patient satisfaction, and medication adherence scores one and six months after discharge were compared between the two groups.

**Result:**

Compared with the control group, the experimental group exhibited reduced PIMs, length of hospital stay, drug costs, and incidence of ADRs (*P* < 0.05). Patient satisfaction of the experimental group was higher than that of the control group (*P* < 0.05). Medication adherence of both groups after discharge was significantly improved compared to that at admission, and the improvement in the experimental group was significantly better than that in the control group, with a statistically significant difference (*P* < 0.05).

**Conclusion:**

Standardized MR services for CHD patients may be conducive to ensuring rational medication and improving clinical effect.

## INTRODUCTION

Coronary atherosclerotic heart disease (hereinafter referred to as “coronary heart disease, CHD”) is a heart disease caused by myocardial ischemia, hypoxia or necrosis as a result of coronary atherosclerosis. Currently, as the population ages, its incidence is rising and it has become one of the main chronic diseases threatening human health. It is estimated that around 11.39 million patients suffer from CHD in China with an increasing prevalence, which causes a huge social and economic burden.^1^-^3^ CHD patients are often complicated by diseases such as hypertension and hyperlipidemia. Due to factors including aging, comorbidities, polypharmacy and poor adherence, various drug-related problems (DRPs) commonly occur in these patients.^4^ Consequently, standardized medication and guidance, an evaluation of patients’ understanding of medication regimens, and regular follow-up visits can help improve their medication poor adherence, optimize medication efficacy, prevent medication errors, reduce medical costs and promote rational medication.

Medication reconciliation (MR)^5^ is a process in which pharmacists understand medication status, obtain a current complete and accurate medication list (i.e., drug names, frequency of administration, dosage, route of administration, and rational use), compare all drugs in current use with medication orders to evaluate their rationality and consistency, provide suggestions for adjusting medication regimens, and collaborate with medical team to adjust unsuitable medication through communication and relevant information review at admission, transfer or discharge of hospitalized patients. It can effectively enhance patient medication adherence, solve DRPs of CHD patients, ensure medication continuity and accuracy, and reduce medication errors and adverse drug reactions/events (ADRs/ADEs), thus ensuring their medical safety.

To better solve the clinical medication-related problems existing in chronic CHD patients, optimize medication regimens, reduce potentially inappropriate medications (PIMs), delay disease progression and further strengthen rational medication, the present study implemented MR in geriatric patients with CHD, as well as summarization and analysis, to evaluate its effect, providing strategic guidance for pharmaceutical care in chronic disease management for CHD patients in the First Affiliated Hospital of Hebei North University.

## METHODS

This was a retrospective study. A total of 200 CHD patients admitted to the Department of Geriatrics in the First Affiliated Hospital of Hebei North University from January 2023 to July 2024 were selected and randomly divided into a control group and an experimental group, with one hundred patients in each group. Eight patients were lost in both groups, and ultimately 192 patients completed this study, including 97 in the control group and 95 in the experimental group.

### Ethical approval:

This study protocol was approved by the Ethics Committee of the First Affiliated Hospital of Hebei North University (No.: K2021092; date: July 21, 2021) and all patients signed the informed consent.

### Inclusion criteria:

- An age > 60 years.
- Patients initially diagnosed with CHD and treated in the Department of Geriatrics of the First Affiliated Hospital of Hebei North University.
- Diagnosis conforming to the guidelines for the diagnosis and treatment of CHD.
- Drugs used ≥ 2 at admission; patients without hearing, language or cognitive impairments.
- Patients who voluntarily participated in the study and were informed.
- And an ability to communicate well with researchers and complete the study according to relevant requirements.

### Exclusion criteria:

- Patients who died during the research period, provided incorrect contact information, or were unable to cooperate during follow-ups.
- Patients with cognitive impairments.
- And patients who were unwilling or unable to continue to cooperate in the research.

The control group was given conventional medication, during which the main therapeutic drugs included heart rhythm-stabilizing, antihypertensive and antiplatelet drugs. On this basis, the experimental group was additionally serviced with MR. To be more specific, within 24 hours after admission or transfer to the Department of Geriatrics, pharmacists obtained a complete and accurate medication list through pharmaceutical care consultation, established a database of basic patient information, and completed “medication education records for CHD patients” and “medication adherence surveys” during hospitalization. After reviewing past medication, PIM use such as medication omission, unsuitable medication and non-standardized medication was analyzed, medication therapy was evaluated, MR was implemented, ADEs and medication-related problems were recorded and sorted by severity, DRPs were identified, and plans to solve problems were established according to the relevant provisions of MR in the *Medical Institution Pharmaceutical Service Practices*^6^, Beers criteria^7^, evidence-based guidelines, medication instructions, *New Materia Medica (17th Edition)*, *Martindale: The Complete Drug Reference (37th Edition)*, as well as patients’ symptoms, signs and relevant laboratory indicators. Follow-ups were conducted one and six months after discharge, including surveys on home-based medication status and medication adherence, case data collection ceased in July 2024.

### Observation indicators:

After treatment, irrational medication, length of hospital stay, drug costs, occurrence of ADRs, patient adherence and satisfaction were compared between the two groups, and statistical analysis was conducted. PIMs: PIMs were evaluated referring to the Beers criteria (2019 edition)^7^ combined with actual clinical statistics mainly based on the following: the criteria for PIM use in elderly patients; the criteria for disease status-related PIM use in elderly patients; medications should be used with caution in elderly patients; drug-drug interactions should be avoided in elderly patients; medications should be avoided or reduced based on the renal function of elderly patients. Various PIM data of the two groups during hospitalization were recorded, and relevant data during hospitalization were compared between the two groups.

Length of hospital stay, drug costs and incidence of ADRs (1) Length of hospital stay and drug costs: The average length of hospital stay and average drug cost were compared between the two groups. (2) The frequency and specific process of ADRs occurring during hospitalization and follow-ups were collected. According to the Naranjo ADR Probability Scale^8^, correlation evaluation was performed, and ADRs judged as “possible”, “probable” or “certain” were included in the statistics. The incidence of ADRs = the frequency of ADRs/the total number of patients in each group × 100%. The incidence of ADRs was compared between the two groups.

### Patient compliance and satisfaction:

### (1) Medication compliance:

Medication adherence was assessed using the 8-item Morisky Medication Adherence Scale (MMAS-8)^9^, and scored based on eight questions, with a total score of eight. The questionnaire was given to the participants for investigation, and then collected in about five minutes. A score of 7-8 indicated high compliance, a score of 6-7 indicated moderate compliance, and a score <6 indicated poor compliance. Medication adherence of the two groups was assessed at admission, one and six months after discharge, respectively, and score changes were compared between the two groups.

### (2) Patient satisfaction:

A satisfaction questionnaire survey was conducted on discharged patients, and their overall satisfaction and basic satisfaction with the therapeutic process were both included in the statistics. The satisfaction was compared between the two groups.

### Statistical analysis:

The data were analyzed using SPSS 21.0 statistical software. The sample size is estimated by 95% confidence interval, measurement data were expressed as (*X̄*±*S*), and enumeration data were repressed by rate (%). Comparisons were performed with the *t* test and χ^2^ test. *P* < 0.05 was considered as statistically significant.

## RESULTS

In this study, a total of 192 patients were surveyed and randomly divided into a control group (n = 97) and an experimental group (n = 95). Comparison of general data between the two groups showed no statistically significant differences (*P* > 0.05), suggesting comparability (Table-I).

**Table-I:** Comparison of general data between the two groups [n (%), *X̄*±*S*].

Item		Control group (n = 97)	Experimental group (n = 95)	P
Gender	Male	63 (64.97)	65 (61.75)	> 0.05
	Female	34 (35.11)	30 (31.29)	> 0.05
Average age (year)		73.05 ± 7.17	74.13 ± 8.03	> 0.05
Smoking history	Yes	59 (60.13)	56 (59.34)	> 0.05
	No	38 (39.82)	39 (40.66)	> 0.05
Other chronic diseases	Yes	73 (70.25)	74 (72.13)	> 0.05
	No	24 (49.37)	21 (21.64)	> 0.05
Number of medications used long-term		5.83 ± 2.01	5.87 ± 2.16	> 0.05

Comparison of PIMs after standardized pharmacist-conducted MR in the experimental group, a total of 54 accumulated PIM issues were collected from the two groups during hospitalization through pharmaceutical care consultations, mainly including unsuitable drug selection, unsuitable drug combination, repeated medication, unsuitable usage and dosage, and medication omission. PIM use was compared between the two groups, revealing a significantly lower rate in the experimental group than in the control group (*P* < 0.05) (Table-II).

**Table-II:** Comparison of PIM use between the two groups [n (%), *X̄*±*S*].

PIM type	Control group (n = 97)	Experimental group (n = 95)	P
Unsuitable drug selection	6 (6.18)	2 (2.05)	
Unsuitable drug combination (including combined use with traditional Chinese medicine and health products)	11 (11.3)	5 (5.26)	
Repeated medication	11 (11.3)	4 (4.21)	
Unsuitable usage and dosage	6 (6.18)	2 (2.05)	
Medication omission/missing	5 (5.15)	0	
Others	2 (2.06)	2 (2.05)	
Total	41 (42.27)	13 (13.68)	p<0.05

Comparison of length of hospital stay, drug costs and incidence of ADRs Comparisons were carried out between the two groups in the average length of hospital stay, average drug cost and incidence of ADRs, which were all significantly lower in the experimental group compared with the control group (*P* < 0.05) (Table-III).

**Table-III:** Comparison of length of hospital stay, drug costs and incidence of ADRs between the two groups (*X̄*±*S*).

Item	Control group (n = 97)	Experimental group (n = 95)	P
Length of hospital stay (d)	15.42±3.79	11.27±5.13	<0.05
Drug costs (yuan)	14766.51±4505.59	13305.87±4022.27	<0.05
Incidence of ADRs [n (%)]	22 (22.68)	6 (6.31)	<0.01

Comparison of medication adherence and patient satisfaction No statistically significant difference was found in medication adherence between the two groups during the early period of hospitalization (*P* > 0.05). One and six months after discharge, medication adherence scores in both groups were significantly improved compared with those before treatment (*P* < 0.05). The improvement in medication adherence scores of the control group was more obvious than that of the experimental group one and six months after discharge (*P* < 0.05). Follow-ups after discharge showed higher patient satisfaction in the experimental group compared with the control group (Table-IV).

**Table-IV:** Comparison of medication adherence and patient satisfaction scores between the two groups during early period of hospitalization, one and six months after discharge (*X̄*±*S*, point).

Time	Group (n)	Medication adherence score	Patient satisfaction [n (%)]
Control group (n=97)	During early period of hospitalization	5.07±0.49	
	1 month after discharge	6.42±0.43^a^	72 (79.38)
	6 months after discharge	6.11±0.37^a^	63 (75.25)
Experimental group (n=95)	During early period of hospitalization	5.23±0.41	
	1 month after discharge	7.52±0.36^ab^	86 (90.52)^b^
	6 months after discharge	7.35±0.47^ab^	80 (84.21)^b^

***Notes:*** Compared with the same group at admission,

a P < 0.05; compared with the control group one and six months after discharge,

b P < 0.05.

## DISCUSSION

In this study, the 192 patients who participated in the survey were randomly divided into a control group and an experimental group. Pharmacists implemented MR services in the experimental group on the basis of conventional clinical treatment. Compared with the control group, the experimental group exhibited significantly reduced PIMs such as irrational drug combination, unsuitable usage and dosage during hospitalization, length of hospital stay, drug costs and incidence of ADRs, and the differences were all statistically significant (*P* < 0.05). In addition, the patient adherence scores of both groups improved significantly one and six months after discharge compared to those during the early period of hospitalization, with statistically significant differences (*P* < 0.05). The inter-group comparison showed that through standardized MR services and follow-ups, the improvement in medication adherence scores and patient satisfaction of the experimental group were significantly superior to those of the control group, presenting statistically significant differences (*P* < 0.05). The above results demonstrate that MR services can improve the rational use of therapeutic drugs for chronic diseases in CHD patients, optimize medication efficacy, and reduce medical costs.^10^

CHD is a common and frequently-occurring disease of the elderly. As the population ages in China, the incidence of CHD is increasing, which has seriously affected the quality of life of the patients. According to the *Chinese Health Statistics Yearbook 2021*, the mortality rate of CHD was 184.17/100,000 in the urban population aged 65 years and above in China, and 216.31/100,000 in the rural population in 2020. Patients with cardiovascular diseases are prone to DRPs during the therapeutic process.^11^ A study^12^ has shown that 30% of CHD patients often have other underlying diseases, and more than 68% of the elderly take ≥ 5 drugs per day. Polypharmacy can lead to DRPs such as medication errors. It has also been demonstrated that each drug addition will increase DRPs by 10%, causing an increased risk of hospitalization.^13^-^15^

Traditional multidisciplinary team specialized in CHD lacks pharmacist-provided pharmaceutical services, resulting in poor medication adherence (such as correct use of multiple drugs, long-term regular medication, etc.), which reduces medication efficacy while increasing acute exacerbations and hospitalizations per year.^16^-^18^ Multiple randomized clinical trials (RCTs) have confirmed that pharmacist-led MR and patient education can reduce inappropriate long-term use of drugs, effectively save medication costs, and benefit high-risk elderly patients with chronic diseases the most in combination interventions, indicating that this pharmaceutical service is a cost-effective strategy.^19^,^20^

The implementation of MR services in medical institutions can help enhance patient satisfaction, improve service connotation, and also reflect the necessity of changing the pharmaceutical care service model in hospitals. Pharmacist-led patient-centered MR services mainly include core elements such as medication review, personal medication records, an action plan for pharmaceutical care, interventions and/or referrals, as well as recording and follow-up.^21^ Establishing a standardized MR process and a pharmaceutical monitoring plan for CHD, as well as emphasizing the cultivation of patients’ proactive management ability in medication, can help improve patient medication adherence, significantly enhance medication safety after discharge, prevent medication errors, reduce the occurrence of ADRs, promote rational clinical medication, and save medical expenses.

### Limitations:

However, this study was only conducted in a third-grade class A hospital, with limited statistical time, scope, and comprehensiveness. For this reason, the sample size needs to be enlarged and multicenter sampling should be adopted in future studies to make the results more reliable.

## CONCLUSIONS

CHD has become one of the most common chronic diseases. During its clinical diagnosis and treatment, patients’ understanding of their own diseases and medication adherence can directly affect medication efficacy. Establishing an MR service model for hospitalized CHD patients based on domestic and international concepts of medication therapy may optimize patients’ medication regimens, promote safe, effective and economical medication, shorten the length of hospital stay, and reduce medical costs. Moreover, it may decrease the incidence of ADRs, and improve patient medication adherence and disease control, providing practical guidance for exploring a novel pharmaceutical care service model for pharmacists in our hospital. Therefore, it is worth promoting and applying in clinical practice.

### Authors’ Contributions:

**MZ:** Concept, literature search and design of study.

**NW** and **XH:** Critical revision of manuscript and responsible for data integrity.

**JY** and **YC:** Data collection, analysis and interpretations.

All authors contributed to the writing of the manuscript, read and approved the final version of the manuscript.
